# Supplementary material for: Transferability of features for neural networks links to adversarial attacks and defences
Source: PLoS One. 2022 Apr 27;17(4):e0266060. doi: 10.1371/journal.pone.0266060 (PMC9045664; doi:10.1371/journal.pone.0266060)
Supplement: S1 File — (PDF) [file pone.0266060.s001.pdf]

S1 Appendix Details About Customised Sub-Imagenet Dataset

Sub-Imagenet is a subset of the Imagenet (ILSVRC 2012) [1] dataset. It is intuitive for us to expect that as the number of classes (N) grows, the decision boundary will become more complicated, causing the classifier to smoothen the representation (Amalgam Proportion) more. Therefore, to prevent this bias, we grouped a subset of 100 existing semantically alike ImageNet classes into 10 distinct super-classes, as described in S1 Table. Our Sub-Imagenet dataset has some desired characteristics for our experiments which are also similar to the CIFAR-10 dataset. These features are:

S1 Table. Description of Super-Classes used in the Sub-ImageNet.

Super-Classes	Training Images	Testing Images	Corresponding Imagenet (ILSVRC 2012) Classes
**Automobile**	12981	500	407, 468, 555, 627, 654, 779, 817, 802, 866, 867
**Ball**	12971	500	429, 430, 522, 574, 722, 746, 768, 805, 852, 890
**Bird**	12990	500	7, 8, 9, 16, 22, 23, 24, 84, 94, 100
**Dog**	12904	500	205, 206, 207, 208, 209, 210, 211, 212, 213, 214
**Feline**	13000	500	283, 284, 285, 286, 287, 288, 289, 290, 291, 292
**Fruit**	12986	500	948, 949, 950, 951, 952, 953, 954, 955, 956, 957
**Insect**	12985	500	300, 301, 302, 303, 304, 305, 306, 307, 308, 309
**Snake**	12758	500	55, 56, 57, 58, 59, 60, 61, 62, 63, 64
**Primate**	12979	500	365, 366, 367, 368, 369, 370, 371, 372, 373, 374
**Vegetable**	12815	500	935, 936, 937, 938, 939, 943, 944, 945, 946, 947
Total	***129359***	***5000***	

1. It is relatively balanced dataset as other datasets used in the experiments. The dataset has a mean of 12937 training images with a standard deviation of 80 images. All super-classes have relatively the same number of images with a minimum of 12758 images for super-class Snake and a maximum of 13000 for super-class Feline. Thus, the samples in the unknown class in our experiments remain relative same.
2. Type of super-classes is similar to CIFAR-10, having six animal classes and four non-animal classes.
3. Abstract Relationships between super-classes also exists similar to the CIFAR-10. The CIFAR-10 have a Cat-Dog and Automobile-Truck relationships in which they are semantically similar. Similarly, our Sub-imagenet also exhibits Dog-Feline and Fruit-Vegetable relationships. These abstract relationships are essential to validate our hypothesis of Amalgam Proportion.

S2 Appendix Details About Standard and Raw Zero-Shot Classifiers

S2 Table shows the classifier accuracy and corresponding loss value on the test dataset of the learned classes. All the classifiers except CapsNet are trained using standard cross-entropy loss. In contrast, CapsNet uses 'margin loss' [2] to train the parameters of the network. As Raw Zero-Shot Classifier, forcefully excludes the images of a class for training, we get the accuracy of the Raw Zero-Shot Classifier on $N-1$ learned classes of the dataset.

S2 Table. Classifier Accuracy (and loss value) on test dataset of the learned classes for different architectures.

Architecture	Standard Classifier	Raw Zero-Shot Classifiers (excluding one class)									
		Fashion MNIST									
		T-Shirt	Trouser	Pullover	Dress	Coat	Sandal	Shirt	Sneaker	Bag	AnkleBoot
**MLP**	88.26% (0.3283)	90.61% (0.2636)	87.73% (0.3440)	91.30% (0.2498)	89.18% (0.2986)	90.96% (0.2606)	88.02% (0.3263)	93.53% (0.1884)	88.57% (0.3241)	87.58% (0.3446)	88.26% (0.3328)
**ConvNet**	90.47% (0.3280)	91.90% (0.2940)	89.43% (0.3556)	90.64% (0.3193)	90.67% (0.3193)	91.70% (0.2933)	88.86% (0.3675)	94.18% (0.2336)	90.20% (0.3309)	90.02% (0.3496)	90.52% (0.3360)
		CIFAR-10									
		Airplane	Automobile	Bird	Cat	Deer	Dog	Frog	Horse	Ship	Truck
**LeNet**	73.86% (0.8223)	74.52% (0.7858)	74.91% (0.7945)	77.33% (0.7075)	79.34% (0.6642)	77.13% (0.7223)	78.32% (0.6990)	75.95% (0.7569)	75.90% (0.7615)	74.46% (0.7946)	75.67% (0.7735)
**VGG**	92.65% (0.5467)	92.85% (0.5370)	92.06% (0.5600)	93.27% (0.5026)	94.43% (0.4660)	92.73% (0.5241)	93.85% (0.4890)	92.82% (0.5400)	92.64% (0.5301)	92.41% (0.5415)	92.60% (0.5442)
**AllConv**	87.93% (0.6823)	88.63% (0.6501)	86.75% (0.7601)	89.07% (0.6313)	90.04% (0.5917)	87.98% (0.6737)	89.81% (0.6147)	87.38% (0.6778)	87.67% (0.7085)	87.35% (0.7034)	87.00% (0.7205)
**NIN**	90.45% (0.5020)	90.92% (0.4752)	90.55% (0.4974)	91.04% (0.4666)	92.84% (0.3962)	91.02% (0.4773)	92.01% (0.4315)	90.77% (0.4646)	90.13% (0.5155)	90.51% (0.5041)	90.26% (0.5068)
**ResNet**	92.58% (0.4685)	92.82% (0.4494)	92.67% (0.4824)	93.42% (0.4328)	94.25% (0.3673)	92.48% (0.4724)	93.75% (0.4119)	92.58% (0.4641)	92.73% (0.4636)	92.53% (0.4740)	92.95% (0.4509)
**DenseNet**	93.97% (0.3643)	94.27% (0.3540)	94.08% (0.3644)	94.20% (0.3341)	95.86% (0.2702)	93.68% (0.3924)	95.15% (0.3054)	94.11% (0.3804)	93.81% (0.3841)	94.07% (0.3627)	94.32% (0.3656)
**WideResNet**	95.02% (0.2705)	94.90% (0.2808)	94.96% (0.2872)	94.96% (0.2761)	96.57% (0.2005)	94.67% (0.3001)	95.98% (0.2318)	94.73% (0.2943)	94.71% (0.2842)	95.01% (0.2844)	94.96% (0.2842)
**CapsNet***	74.74% (0.2017)	75.20% (0.1953)	74.43% (0.2022)	76.74% (0.1878)	77.35% (0.1875)	76.86% (0.1877)	77.33% (0.1838)	75.92% (0.1949)	74.71% (0.2004)	74.92% (0.2001)	74.43% (0.2015)
		Sub-Imagenet									
		Automobile	Ball	Bird	Dog	Feline	Fruit	Insect	Snake	Primate	Vegetable
**InceptionV3**	94.06% (0.1907)	94.22% (0.1968)	95.00% (0.1686)	93.91% (0.2787)	93.93% (0.1977)	93.60% (0.1997)	95.11% (0.1702)	94.60% (0.2031)	94.40% (0.1870)	94.33% (0.1937)	94.66% (0.3634)
**ResNet-50**	92.58% (0.2590)	91.04% (1.7212)	91.08% (1.0540)	92.91% (0.3529)	92.15% (0.3986)	31.40% (1.8681)	95.64% (0.1647)	94.17% (0.4806)	92.66% (0.2975)	94.02% (0.2278)	94.68% (0.2891)

S3 Appendix Details About Adversarial Defences

All the adversarial defences used in the article have been evaluated using Adversarial Robustness 360 Toolbox (ART v1.2.0) [3]. S3 Table describes the defence parameters used for the evaluated adversarial defences. S4 Table shows the classifier accuracy and corresponding loss value on the test dataset of the learned classes for various adversarial defences. All the classifiers except CapsNet use standard cross-entropy loss, while CapsNet uses margin loss [2]. As Raw Zero-Shot Classifier, forcefully excludes the images of a class for training, we get the accuracy of the Raw Zero-Shot Classifier on $N-1$ learned classes of the dataset.

S3 Table. Description of Adversarial Defence Parameters.

Defence	Parameters
**Gaussian Augmentation (G Aug)**	$\sigma = 1.0$
**Feature Squeezing (FS)**	bit depth = 5
**Spatial Smoothing (SS)**	window size = 3
**Label Smoothing (LS)**	max value = 0.9
**Thermometer Encoding (TE)**	num space = 16
**Adversarial Training (AT)**	Attack: Projected Gradient Descent (PGD)<br>Attack Parameters: norm = L_∞, $\epsilon = 8$, $\epsilon_{\text{step}} = 2$, iterations = 10

S4 Appendix Details About Adversarial Attacks

All the adversarial attacks used in the article have been evaluated using Adversarial Robustness 360 Toolbox (ART v1.2.0) [3]. We evaluated the test samples of Fashion MNIST, CIFAR-10 and Sub Imagenet datasets for the adversarial attacks on standard classifiers. We fixed the parameters of the attacks evaluated, and S5 Table describes the attack parameters used for the evaluated adversarial attacks. S6 Table shows the adversarial accuracy and Mean L_2 Score for each classifier and adversarial attack pair. Here, Adversarial Accuracy corresponds to the percentage of adversarial images

S4 Table. Classifier Accuracy on test dataset of the learned classes for different architectures.

Architecture	Standard Classifier	Raw Zero-Shot Classifiers (excluding one class) Airplane	Automobile	Bird	Cat	Deer	Dog	Frog	Horse	Ship	Truck
					Gaussian Augmentation (G Aug)						
**LeNet**	76.05% (0.7795)	76.35% (0.7736)	73.73% (0.8315)	77.15% (0.7151)	79.43% (0.6736)	76.57% (0.7396)	77.11% (0.7175)	76.20% (0.7579)	75.18% (0.7836)	75.91% (0.7763)	75.27% (0.7999)
**VGG**	92.64% (0.5276)	93.01% (0.5123)	92.58% (0.5392)	93.63% (0.4749)	94.63% (0.4425)	92.65% (0.5289)	94.21% (0.4566)	92.78% (0.5272)	92.77% (0.5074)	92.53% (0.5505)	92.96% (0.5291)
**AllConv**	87.74% (0.7554)	88.68% (0.6927)	86.95% (0.7693)	89.00% (0.6644)	90.33% (0.5983)	88.58% (0.6783)	90.27% (0.6190)	86.72% (0.8040)	88.12% (0.7196)	87.54% (0.7415)	86.88% (0.8059)
**NIN**	91.14% (0.4845)	91.31% (0.4728)	91.31% (0.4910)	91.96% (0.4600)	93.64% (0.3706)	91.31% (0.4791)	92.30% (0.4249)	91.04% (0.4803)	91.20% (0.4926)	91.02% (0.4811)	91.05% (0.4790)
**ResNet**	93.02% (0.4340)	93.33% (0.4232)	92.77% (0.4151)	93.57% (0.3862)	94.81% (0.3136)	93.13% (0.4103)	94.20% (0.3320)	92.76% (0.4058)	93.06% (0.4214)	92.63% (0.4466)	92.94% (0.4281)
**DenseNet**	94.56% (0.2963)	94.65% (0.2949)	94.81% (0.3136)	94.94% (0.2710)	96.08% (0.2036)	94.23% (0.3092)	95.80% (0.2255)	94.36% (0.2969)	94.47% (0.3068)	94.34% (0.3127)	95.01% (0.2850)
**WideResNet**	95.05% (0.2580)	95.24% (0.2529)	95.20% (0.2701)	95.42% (0.2406)	96.55% (0.1848)	95.22% (0.2532)	96.08% (0.2078)	.% (0.)	94.86% (0.2673)	95.14% (0.2717)	95.25% (0.2485)
**CapsNet**	76.29% (0.1938)	77.08% (0.1833)	76.00% (0.1932)	78.20% (0.1771)	76.87% (0.1913)	78.21% (0.1790)	79.52% (0.1712)	77.21% (0.1864)	76.73% (0.1879)	75.87% (0.1926)	76.28% (0.1877)
					Feature Squeezing (FS)						
**LeNet**	73.92% (0.8218)	74.45% (0.7856)	74.94% (0.7945)	77.26% (0.7077)	79.38% (0.6630)	76.94% (0.7228)	78.22% (0.6987)	76.05% (0.7564)	75.95% (0.7608)	74.36% (0.7954)	75.61% (0.7736)
**VGG**	92.65% (0.5470)	92.77% (0.5377)	92.06% (0.5614)	93.25% (0.5026)	94.42% (0.4662)	92.67% (0.5250)	93.87% (0.4890)	92.88% (0.5402)	92.66% (0.5303)	92.52% (0.5424)	92.57% (0.5449)
**AllConv**	87.93% (0.6812)	88.63% (0.6488)	86.78% (0.7609)	89.00% (0.6310)	90.06% (0.5915)	87.87% (0.6744)	89.77% (0.6151)	87.46% (0.6768)	87.50% (0.7090)	87.36% (0.7027)	86.97% (0.7210)
**NIN**	90.54% (0.5032)	90.96% (0.4769)	90.52% (0.4995)	91.05% (0.4674)	92.80% (0.3966)	91.03% (0.4778)	92.03% (0.4321)	90.67% (0.4650)	90.12% (0.5162)	90.47% (0.5057)	90.18% (0.5090)
**ResNet**	92.54% (0.4707)	92.72% (0.4499)	92.50% (0.4802)	93.47% (0.4332)	94.08% (0.3690)	92.34% (0.4756)	93.82% (0.4114)	92.62% (0.4646)	92.85% (0.4656)	92.35% (0.4749)	92.98% (0.4519)
**DenseNet**	93.92% (0.3662)	94.23% (0.3553)	93.98% (0.3661)	94.35% (0.3338)	95.81% (0.2712)	93.72% (0.3938)	95.10% (0.3061)	94.08% (0.3799)	93.82% (0.3843)	94.02% (0.3665)	94.21% (0.3648)
**WideResNet**	94.96% (0.2722)	94.92% (0.2815)	94.81% (0.2876)	94.92% (0.2775)	96.58% (0.2001)	94.51% (0.3015)	95.93% (0.2332)	94.50% (0.2960)	94.77% (0.2859)	94.94% (0.2855)	95.00% (0.2870)
**CapsNet**	74.73% (0.2016)	75.26% (0.1952)	74.31% (0.2022)	76.80% (0.1879)	77.33% (0.1875)	76.80% (0.1877)	77.38% (0.1838)	75.81% (0.1947)	74.66% (0.2004)	74.88% (0.2001)	74.36% (0.2016)
					Spatial Smoothing (SS)						
**LeNet**	70.01% (0.9215)	69.64% (0.9056)	69.58% (0.9161)	73.24% (0.8163)	74.84% (0.7866)	71.96% (0.8391)	72.98% (0.8421)	71.16% (0.8789)	70.32% (0.9043)	69.44% (0.9182)	70.45% (0.8919)
**VGG**	83.32% (0.9154)	83.84% (0.9086)	83.13% (0.9237)	85.28% (0.8036)	86.71% (0.7975)	83.46% (0.8935)	86.31% (0.8000)	83.20% (0.9398)	83.75% (0.8702)	83.96% (0.8852)	83.71% (0.8982)
**AllConv**	81.31% (0.8986)	81.53% (0.9149)	79.12% (1.0347)	82.81% (0.8419)	82.75% (0.9173)	81.23% (0.8904)	83.66% (0.8442)	80.65% (0.9401)	81.84% (0.8994)	81.70% (0.8861)	82.55% (0.8602)
**NIN**	84.57% (0.7081)	85.11% (0.6947)	84.71% (0.7023)	85.84% (0.6542)	87.78% (0.5963)	84.86% (0.6872)	87.71% (0.5978)	84.94% (0.7071)	84.82% (0.7129)	85.16% (0.6877)	85.00% (0.6977)
**ResNet**	77.19% (1.4403)	74.57% (1.6944)	78.64% (1.3998)	79.53% (1.3088)	82.51% (1.0585)	76.51% (1.6006)	79.72% (1.3452)	78.96% (1.2993)	79.78% (1.2429)	77.46% (1.4125)	78.62% (1.3955)
**DenseNet**	78.59% (1.1361)	76.18% (1.4147)	77.20% (1.3156)	79.07% (1.2178)	80.38% (1.0384)	77.88% (1.1653)	80.24% (1.0934)	78.75% (1.2140)	79.35% (1.1429)	77.67% (1.2486)	79.26% (1.1859)
**WideResNet**	76.93% (1.1204)	77.41% (1.0795)	78.77% (0.9918)	77.33% (1.0777)	79.92% (0.9149)	77.16% (1.1398)	79.94% (0.9821)	77.77% (1.0508)	77.04% (1.1066)	77.62% (1.0670)	78.72% (0.9773)
**CapsNet**	72.58% (0.2213)	73.32% (0.2139)	71.55% (0.2226)	74.23% (0.2051)	74.68% (0.2072)	73.97% (0.2082)	74.52% (0.2034)	72.96% (0.2137)	72.23% (0.2184)	72.80% (0.2170)	71.96% (0.2204)
					Label Smoothing (LS)						
**LeNet**	75.15% (0.8138)	73.75% (0.8275)	74.04% (0.8233)	77.30% (0.7345)	78.94% (0.7011)	76.04% (0.7668)	77.65% (0.7297)	75.88% (0.7764)	74.70% (0.7967)	74.71% (0.8141)	74.93% (0.8047)
**VGG**	92.63% (0.5205)	92.94% (0.4901)	92.06% (0.5153)	93.25% (0.4723)	94.51% (0.4390)	92.46% (0.4896)	93.91% (0.4609)	92.46% (0.5008)	92.51% (0.4922)	92.56% (0.5025)	92.52% (0.5034)
**AllConv**	89.03% (0.5349)	89.23% (0.5044)	86.54% (0.5973)	89.37% (0.5091)	90.24% (0.4730)	89.06% (0.5272)	89.97% (0.4995)	88.51% (0.5381)	88.00% (0.5374)	87.85% (0.5568)	88.46% (0.5402)
**NIN**	90.28% (0.4102)	90.78% (0.3986)	90.13% (0.4148)	91.26% (0.3819)	92.95% (0.3297)	90.97% (0.3984)	92.02% (0.3582)	90.74% (0.3921)	90.56% (0.4020)	90.17% (0.4157)	90.34% (0.4085)
**ResNet**	92.48% (0.4126)	93.01% (0.3910)	92.54% (0.4036)	93.42% (0.3724)	94.38% (0.3399)	92.81% (0.3949)	94.02% (0.3500)	92.58% (0.4010)	92.06% (0.4166)	93.04% (0.3977)	92.41% (0.4048)
**DenseNet**	94.08% (0.3712)	94.70% (0.3424)	94.10% (0.3616)	94.85% (0.3405)	95.47% (0.3067)	94.03% (0.3606)	95.31% (0.3180)	94.50% (0.3485)	93.63% (0.3738)	94.30% (0.3620)	94.20% (0.3626)
**WideResNet**	95.12% (0.2954)	95.33% (0.2935)	95.03% (0.3014)	95.44% (0.2849)	96.61% (0.2486)	94.97% (0.3018)	95.84% (0.2646)	95.06% (0.2998)	95.21% (0.2957)	95.14% (0.2982)	95.18% (0.2914)
**CapsNet**	74.24% (0.2115)	75.95% (0.1979)	74.95% (0.2034)	77.83% (0.1849)	78.40% (0.1863)	72.74% (0.2220)	77.88% (0.1865)	76.48% (0.1978)	76.35% (0.2004)	75.35% (0.2004)	74.84% (0.2072)
					Thermometer Encoding (TE)						
**LeNet**	65.73% (1.0599)	64.36% (1.0869)	64.21% (1.0705)	68.68% (0.9427)	68.63% (0.9557)	67.84% (0.9763)	67.95% (0.9905)	64.60% (1.0635)	66.60% (1.0139)	65.70% (1.0272)	65.92% (1.0513)
**VGG**	84.01% (0.8730)	82.77% (0.9430)	83.72% (0.8934)	84.96% (0.8305)	86.97% (0.7566)	85.01% (0.8250)	85.74% (0.8114)	84.62% (0.8435)	83.78% (0.8753)	83.94% (0.8783)	81.83% (0.9777)
**AllConv**	77.71% (1.1347)	78.00% (1.0777)	76.86% (1.1854)	79.41% (1.0531)	81.21% (0.9174)	79.24% (0.9883)	80.01% (1.0531)	78.84% (1.0680)	78.40% (1.0330)	77.47% (1.0763)	77.34% (1.1180)
**NIN**	81.75% (0.8457)	82.80% (0.8031)	81.78% (0.8305)	83.48% (0.7702)	84.91% (0.7120)	82.80% (0.7990)	84.03% (0.7526)	82.00% (0.8240)	82.00% (0.8579)	81.38% (0.8481)	81.27% (0.8554)
**ResNet**	83.04% (1.0909)	83.55% (1.0777)	82.65% (1.1141)	84.56% (0.9993)	86.14% (0.8782)	83.81% (1.0596)	85.31% (0.9196)	83.44% (1.0720)	82.66% (1.1020)	82.91% (1.1110)	83.06% (1.1231)
**DenseNet**	85.08% (0.9698)	85.93% (0.9420)	84.73% (0.9839)	86.97% (0.8584)	88.05% (0.7693)	86.27% (0.9042)	87.41% (0.8407)	85.08% (0.9649)	85.33% (0.9369)	85.18% (0.9697)	85.93% (0.9338)
**WideResNet**	86.55% (0.7288)	86.33% (0.7479)	86.14% (0.7623)	87.78% (0.6851)	89.26% (0.5762)	86.83% (0.7120)	88.42% (0.6256)	86.48% (0.7444)	86.40% (0.7646)	86.16% (0.7493)	86.48% (0.7286)
**CapsNet**	32.45% (0.6018)	25.58% (0.6569)	60.31% (0.2874)	62.42% (0.2744)	49.08% (0.4128)	41.34% (0.5214)	60.75% (0.2868)	47.27% (0.4277)	41.77% (0.4795)	59.63% (0.2926)	44.30% (0.4665)
					Adversarial Training (AT)						
**LeNet**	60.87% (1.2041)	60.47% (1.1789)	61.47% (1.1583)	64.53% (1.0746)	65.66% (1.0460)	63.73% (1.0766)	65.13% (1.0730)	61.81% (1.1341)	61.91% (1.1414)	61.13% (1.1649)	61.62% (1.1502)
**VGG**	82.20% (0.7734)	83.35% (0.7194)	84.45% (0.7192)	84.96% (0.6834)	87.33% (0.6344)	85.11% (0.6706)	86.97% (0.6412)	82.95% (0.7273)	83.28% (0.7395)	83.80% (0.7537)	84.61% (0.7087)
**AllConv**	80.99% (0.7222)	81.23% (0.7077)	78.63% (0.7888)	82.55% (0.6645)	84.20% (0.6321)	81.42% (0.6756)	83.30% (0.6543)	80.45% (0.7303)	80.05% (0.7271)	79.95% (0.7501)	80.84% (0.7262)
**NIN**	83.98% (0.6079)	84.73% (0.5774)	83.21% (0.6130)	85.57% (0.5305)	87.57% (0.4820)	85.33% (0.5294)	86.92% (0.5127)	84.83% (0.5729)	84.38% (0.5866)	84.26% (0.5928)	85.41% (0.5868)
**ResNet**	82.53% (0.6259)	81.63% (0.6726)	83.72% (0.6039)	84.27% (0.5981)	86.56% (0.5302)	82.97% (0.6103)	84.45% (0.6006)	83.21% (0.6110)	82.00% (0.6728)	82.12% (0.6802)	79.30% (0.7027)
**DenseNet**	85.40% (0.5815)	84.95% (0.6055)	84.08% (0.6355)	86.34% (0.5379)	88.14% (0.4942)	85.91% (0.5378)	88.27% (0.5096)	85.16% (0.5934)	82.91% (0.8311)	84.16% (0.6158)	83.80% (0.6019)
**WideResNet**	84.67% (0.8057)	84.90% (0.7483)	84.11% (0.8626)	85.78% (0.7780)	88.43% (0.6124)	85.30% (0.8425)	87.57% (0.6585)	85.08% (0.7759)	84.50% (0.8096)	83.55% (0.8256)	84.35% (0.8034)
**CapsNet**	65.97% (0.2746)	65.75% (0.2678)	64.92% (0.2694)	69.87% (0.2457)	71.95% (0.2377)	70.55% (0.2425)	52.71% (0.3352)	62.71% (0.2842)	67.48% (0.2605)	65.63% (0.2697)	67.58% (0.2579)

misclassified by a standard classifier. While Mean L_2 Score corresponds to the Mean L_2 norm of the perturbation in the adversarial image.

Attack	For Fashion MNIST	For CIFAR-10 and Sub Imagenet
**FGM**	norm = L_∞, $\epsilon = 0.3$, $\epsilon_{\text{step}} = 0.01$	norm = L_∞, $\epsilon = 8$, $\epsilon_{\text{step}} = 2$
**BIM**	norm = L_∞, $\epsilon = 0.3$, $\epsilon_{\text{step}} = 0.01$, iterations = 80	norm = L_∞, $\epsilon = 8$, $\epsilon_{\text{step}} = 2$, iterations = 10
**PGD**	norm = L_∞, $\epsilon = 0.3$, $\epsilon_{\text{step}} = 0.01$, iterations = 40	norm = L_∞, $\epsilon = 8$, $\epsilon_{\text{step}} = 2$, iterations = 20
**DF**	iterations = 100, $\epsilon = 0.02$	iterations = 100, $\epsilon = 0.000001$
**NF**	iterations = 100, eta = 0.375	iterations = 100, eta = 0.01

S5 Table. Description of Adversarial Attack Parameters

S5 Appendix More Visualisations of Davies–Bouldin Metric (DBM)

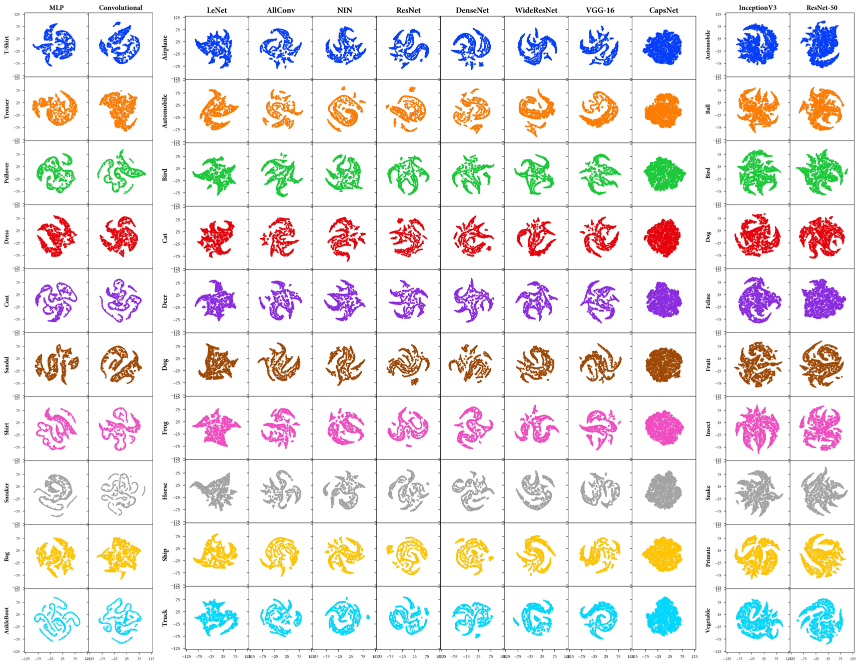

S1 Fig. Visualisation of the Davies–Bouldin Metric (DBM) results for vanilla classifiers using t-Distributed Stochastic Neighbour Embedding (t-SNE). Each row represents a classifier trained with a label excluded whose projection is visualised.

S1-S3 Figs shows visualization of Davies–Bouldin Metric (DBM) metric using other manifold visualisations such as, t-Distributed Stochastic Neighbour Embedding

S6 Table. Adversarial Accuracy and Mean L_2 Score for each classifier and adversarial attack pair.

Classifier	Adversarial Accuracy (in %)					Mean L_2 Score				
	FGM	BIM	PGD	DF	NF	FGM	BIM	PGD	DF	NF
**Fashion MNIST**										
**MLP**	91.08	91.29	91.29	27.16	25.39	210.73	638.83	638.83	309.41	289.28
**ConvNet**	86.89	89.20	89.18	23.63	22.67	306.25	669.56	665.76	314.81	263.65
**CIFAR-10**										
**LeNet**	84.58	89.12	89.25	31.70	84.12	152.37	345.27	357.34	132.32	49.61
**VGG**	82.79	94.97	94.99	65.08	92.43	181.29	321.86	329.96	651.65	77.01
**AllConv**	67.09	69.11	69.11	51.46	61.86	155.95	273.90	274.15	487.46	61.05
**NIN**	72.49	74.26	74.26	59.94	66.76	140.46	216.97	216.96	492.90	54.78
**ResNet**	52.75	55.41	55.41	58.71	54.39	124.70	164.64	164.64	458.57	51.56
**DenseNet**	50.78	52.11	52.11	60.83	50.81	120.03	160.34	160.38	478.03	53.89
**WideResNet**	69.59	89.42	89.44	60.10	82.73	159.88	208.44	208.49	613.14	63.13
**CapsNet**	70.02	82.23	84.46	87.40	90.04	208.89	361.63	370.90	258.08	1680.83
**Sub-Imagenet**										
**InceptionV3**	85.76	87.24	87.24	86.94	58.44	796.53	1204.01	1204.01	609.54	319.73
**ResNet-50**	85.74	86.72	86.72	84.78	60.84	826.06	1264.30	1264.34	633.30	336.80

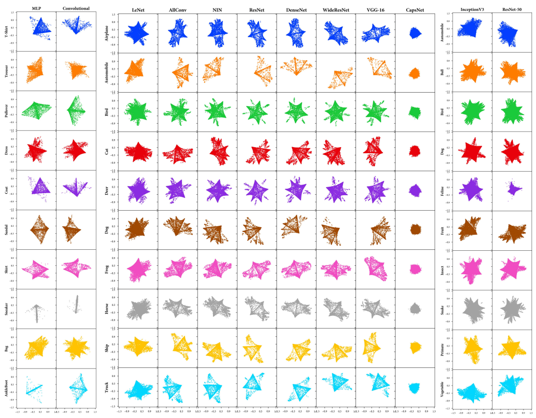

S2 Fig. Visualisation of the Davies–Bouldin Metric (DBM) results for vanilla classifiers using Multi Dimensional Scaling (MDS). Each row represents a classifier trained with a label excluded whose projection is visualised.

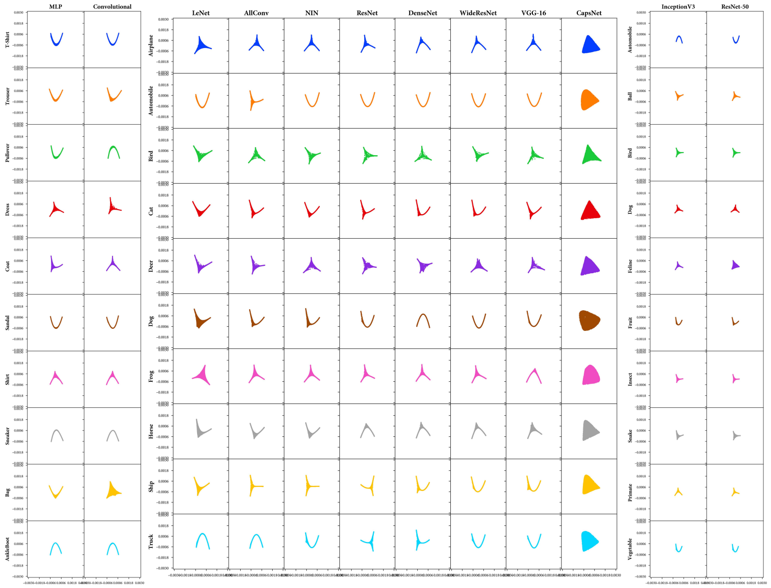

S3 Fig. Visualisation of the Davies–Bouldin Metric (DBM) results for vanilla classifiers using Spectral Embedding (SE). Each row represents a classifier trained with a label excluded whose projection is visualised.

(t-SNE) [4], Multi-dimensional Scaling (MDS) [5], and Spectral Embedding (SE) [6] respectively. t-SNE tries to model similar data points in higher-dimensional space through small pairwise distances in lower-dimensional space. In other words, it tries to minimise the Kullback–Leibler divergence between the two distributions of points in the map. MDS seeks a lower-dimensional representation of the data in which the distances respect well the distances in the original high-dimensional space. SE is a non-linear embedding that finds a lower-dimensional representation of the sample points using a spectral decomposition of the Laplacian Eigenmaps. It is to be noted that Isomap (3) t-SNE (S1 Fig), MDS (S2 Fig), and SE (S3 Fig) are different visualisations for the same feature space. The idea for having these visualisations is to investigate whether the cluster for the unknown class can be segregated into one or more different classes. In other words, we try to investigate visually whether there exists a single combination of Amalgam Proportion for the unknown class.

The projections (S1-S3 Figs) of CapsNet is uniform and dense. At the same time, the other networks have more scattered non-uniform projections. The non-uniform projection, which can be split into multiple clusters, of the other networks might suggest that the learned representation is not continuous/homogeneous enough. Interestingly, LeNet has more dense and uniform projections than other static neural networks, further suggesting the better transferability-of-features of the LeNet. These results are in accordance with the previous experiments on transferability-of-features.

Another way to verify this interpretation is to look at the gaps in the projections, which is observing the behaviour of different data points. For CapsNet, even if we form clusters to have different classes, the gaps between the classes will be too small relative to other architectures. While for the other architectures, some points can form their separate cluster and be termed as a different class. Hence, for these architectures, it can have one or more different Amalgam proportions for the same unknown class, which

contradicts our hypothesis that there should exist only a single Amalgam proportion for a single unknown class. Note that, this dense projection does not necessarily mean that the unknown class has converged to a single known class. It gives a visualisation that the Amalgam Proportion of the unknown class is similar.

References

1. Russakovsky O, Deng J, Su H, Krause J, Satheesh S, Ma S, et al. Imagenet large scale visual recognition challenge. International journal of computer vision. 2015;115(3):211–252.
2. Sabour S, Frosst N, Hinton GE. Dynamic routing between capsules. In: Advances in neural information processing systems; 2017. p. 3856–3866.
3. Nicolae MI, Sinn M, Tran MN, Buesser B, Rawat A, Wistuba M, et al. Adversarial Robustness Toolbox v1.1.0. CoRR. 2018;1807.01069.
4. Maaten Lvd, Hinton G. Visualizing data using t-SNE. Journal of Machine Learning Research. 2008;9(Nov):2579–2605.
5. Kruskal JB. Multidimensional scaling by optimizing goodness of fit to a nonmetric hypothesis. Psychometrika. 1964;29(1):1–27.
6. Belkin M, Niyogi P. Laplacian eigenmaps for dimensionality reduction and data representation. Neural computation. 2003;15(6):1373–1396.
