# Supplementary material for: Transferability of features for neural networks links to adversarial attacks and defences
Source: PLoS One. 2022 Apr 27;17(4):e0266060. doi: 10.1371/journal.pone.0266060 (PMC9045664; doi:10.1371/journal.pone.0266060)
Supplement: Attachment — Submitted filename: Rebuttal - Raw Zero-Shot.pdf [file pone.0266060.s002.pdf]

Dear Academic Editor and Reviewers,

We thank the reviewers for their constructive feedback on our article. We have revised our article according to the feedback and below is our detailed response to the reviews and journal requirements.

Reviewer #1: The section numbers to be mentioned and it is confuse to identify the sections and subsections. Discuss about the remaining sections in the introduction.

The readability of the article was improved by mentioning section numbers and discussing the sections in the introduction according to the feedback.

Reviewer #1: The literature study extended with the recently published articles.

The literature study is revised to discuss some recently published articles in the related fields.

Reviewer #1: Discuss the reasons to achieve the superior performance of the proposed models.

Despite varying types of adversarial attacks, adversarial defences and neural network architectures, our experiments suggest that models which have better transferability-of-features as measured with our metrics were more robust to adversarial attacks. The transferability-of-features for a few interesting models are discussed in Section 8 of the article. Further, a hypothesis of the effect of dataset bias on the transferability-of-features is presented in Section 9 of the article.

Reviewer #1: How about the computational time complexity of the proposed model?

Our approach follows a leave-one-out strategy to exclude a class keeping all other parameters the same as the standard model. Therefore, the evaluation of a single class has the same computational time complexity as training the model with other classes. Since the model is trained on "*n-1*" classes instead of "*n*" classes. However, evaluating all the classes of the dataset requires "*n*" pieces of training, where "*n*" is the number of classes in the dataset.

Reviewer #1: Discuss the limitations of the model.

The technical difficulty and limitations of the proposed scheme are now discussed in the revised Section 4 Experimental Design.

Reviewer #2: Literature review section can be included to discuss works done so far related to the problem.

The literature study is revised to discuss some recently published articles in the related fields.

Reviewer #2: Abstract can conclude with qualitative results of the proposed work.

Thank you for pointing this out. To improve our abstract, we have included the qualitative inference from our experiments.

Reviewer #2: Overall grammar has to be checked

Our article is revised and has been corrected for grammatical and typing mistakes.

Journal Requirement: Please ensure that your manuscript meets PLOS ONE's style requirements, including those for file naming.

The manuscript is checked and it is ensured that the manuscript follows PLOS ONE's style requirement.

2. We noticed you have some minor occurrence of overlapping text with the following previous publication(s), which needs to be addressed:
- https://arxiv.org/abs/1906.06627
In your revision ensure you cite all your sources (including your own works), and quote or rephrase any duplicated text outside the methods section. Further consideration is dependent on these concerns being addressed.

The article "*https://arxiv.org/abs/1906.06627*" is a preprint version of this submitted article in arXiv. Once the final version of this submitted article is accepted, the preprint version on arXiv will be updated.

Journal Requirement: Please remove any funding-related text from the manuscript and let us know how you would like to update your Funding Statement. Currently, your Funding Statement reads as follows:
"DV, JST-Act-I Grant Number JP-50243, https://www.jst.go.jp/kisoken/act-i/index.html
DV, JSPS KAKENHI Grant Number JP20241216, https://www.jsps.go.jp/english/e-grants/"
Please include your amended statements within your cover letter; we will change the online submission form on your behalf.

Funding-related text is removed from the manuscript. Further, we would like our funding statement to be read as follows,

"*Danilo Vasconcellos Vargas, JST-Act-I Grant Number JP-50243,*
https://www.jst.go.jp/kisoken/act-i/index.html

Danilo Vasconcellos Vargas, JSPS KAKENHI Grant Number JP20241216,
https://www.jsps.go.jp/english/e-grants/"

Kindly amend the statements in the submission.

Journal Requirements: We note that Figures 1 and 2 in your submission contain copyrighted images. We require you to either (1) present written permission from the copyright holder to

publish these figures specifically under the CC BY 4.0 license, or (2) remove the figures from your submission.

The figures are updated with no-copyrighted images. Therefore, we are removing the figures with the copyrighted images.

Journal Requirement: Please review your reference list to ensure that it is complete and correct.

The reference list was reviewed and none of the references are retracted.

Yours faithfully

Shashank Kotyan
Moe Matsuki
Danilo Vasconcellos Vargas
